# Senescence-Associated Checkpoint Gene Dysregulation in Established Osteoarthritis: Integrated Transcriptomic Analysis and In Vitro Evaluation of CDK6 and WEE1

**DOI:** 10.3390/biomedicines14091914

**Published:** 2026-08-26

**Authors:** Chang-Sheng Liao, Yu-Can Ju, Min-Xiao Wang, Cheng Long, Feng-Jun Lan

**Affiliations:** 1 Department of Orthopaedics, West China Hospital of Sichuan University, Chengdu 610041, China; 15603441409@163.com (C.-S.L.); juyucan@163.com (Y.-C.J.); 2 Department of Graduate School, Graduate Student Department of Changzhi Medical College, Changzhi 046000, China; 15835558567@163.com

**Keywords:** osteoarthritis, established osteoarthritis, cellular senescence, checkpoint-related genes, CDK6, WEE1, transcriptomic integration

## Abstract

**Background/Objectives**: Osteoarthritis (OA) is a whole-joint disease, and cellular senescence is one of several processes associated with cartilage degeneration. This study aimed to identify senescence-associated differentially expressed genes in established OA and to examine checkpoint-related candidates without assuming a causal checkpoint-imbalance mechanism. **Methods**: Five Gene Expression Omnibus (GEO) datasets spanning articular-cartilage tissue and primary cartilage-derived chondrocytes (GSE57218, GSE117999, GSE114007, GSE246425, and GSE169077) were integrated as a training cohort; the meniscus dataset GSE98918 was reserved as an independent cross-tissue validation cohort. OA-associated differentially expressed genes (DEGs) were intersected with CELLAGE genes. Enrichment, protein–protein interaction, transcription-factor, competing endogenous RNA, drug-enrichment, and molecular-docking analyses were performed. CDK6 and WEE1 expression was evaluated in IL-1β-treated human C28/I2 chondrocytes by RT-qPCR and representative Western blotting. **Results**: Forty-one senescence-associated DEGs were identified, and seven network-central genes (CDKN1A, CDK6, WEE1, NFKB2, ID1, RBL2, and IGFBP7) were prioritized. CDKN1A, CDK6, and WEE1 were reduced in OA-associated meniscal samples in GSE98918; within-dataset ROC analyses yielded AUCs of 0.931, 0.882, and 0.792, respectively. In IL-1β-treated C28/I2 cells, WEE1 mRNA decreased whereas CDK6 mRNA increased; representative immunoblots showed concordant qualitative trends. Berberine- and folic acid-related docking findings were computational only. **Conclusions**: Checkpoint-related gene expression is associated with senescence-linked transcriptomic changes in established OA. The discordant CDK6 results between clinical tissue datasets and an acute inflammatory cell model do not establish stage-dependent regulation. The present data also do not demonstrate p53-mediated CDKN1A activation, checkpoint failure, cell-cycle arrest, or cellular senescence; these hypotheses require dedicated functional experiments.

## 1. Introduction

Osteoarthritis (OA) is a multifactorial disease of the joint as an organ rather than an isolated disorder of articular cartilage. Its pathological spectrum includes articular-cartilage degeneration, meniscal degeneration, subchondral-bone remodeling, synovial-membrane inflammation, and inflammatory and metabolic changes in the infrapatellar fat pad [1,2,3,4,5,6]. Aging is an important risk factor, but OA may also follow joint injury, mechanical overload, obesity, metabolic dysfunction, and other non-age-related insults [3,5,7,8,9].

Cellular senescence is increasingly implicated in OA progression, alongside age-related matrix changes such as advanced glycation end-product accumulation [10,11,12,13,14]. Senescent chondrocytes show persistent cell-cycle arrest and may develop a senescence-associated secretory phenotype that amplifies inflammation and matrix catabolism. Nevertheless, senescence is heterogeneous, and transcriptomic enrichment for senescence-related genes is not equivalent to functional demonstration of senescence. Mechanical stress, oxidative stress, mitochondrial dysfunction, and inflammatory cytokines may predominate at different points in disease development.

Transcriptomic intersection strategies have been used to prioritize senescence-associated genes in OA and other disease contexts [15,16]. Such analyses are useful for candidate generation but remain dependent on dataset composition, annotation resources, and downstream functional confirmation.

Cell-cycle regulators such as CDKN1A, CDK6, and WEE1 are biologically relevant to stress responses, proliferation, and checkpoint control [17,18,19,20,21,22]. However, expression changes in bulk cartilage cannot by themselves establish activation of a p53-CDKN1A pathway, checkpoint failure, or a specific disease stage. Clinical cartilage datasets frequently represent established or advanced OA, whereas acute cytokine exposure in cultured chondrocytes model only a limited inflammatory context.

We therefore integrated multiple OA-related cartilage and cartilage-derived transcriptomic cohorts to identify senescence-associated DEGs and checkpoint-related candidates, used the meniscus dataset GSE98918 exclusively for independent cross-tissue validation, and examined CDK6 and WEE1 in an IL-1β-treated human chondrocyte model. The study was designed as a hypothesis-generating association analysis; it did not test a causal checkpoint-imbalance mechanism or stage-dependent CDK6 dynamics.

## 2. Materials and Methods

**Figure 1:**
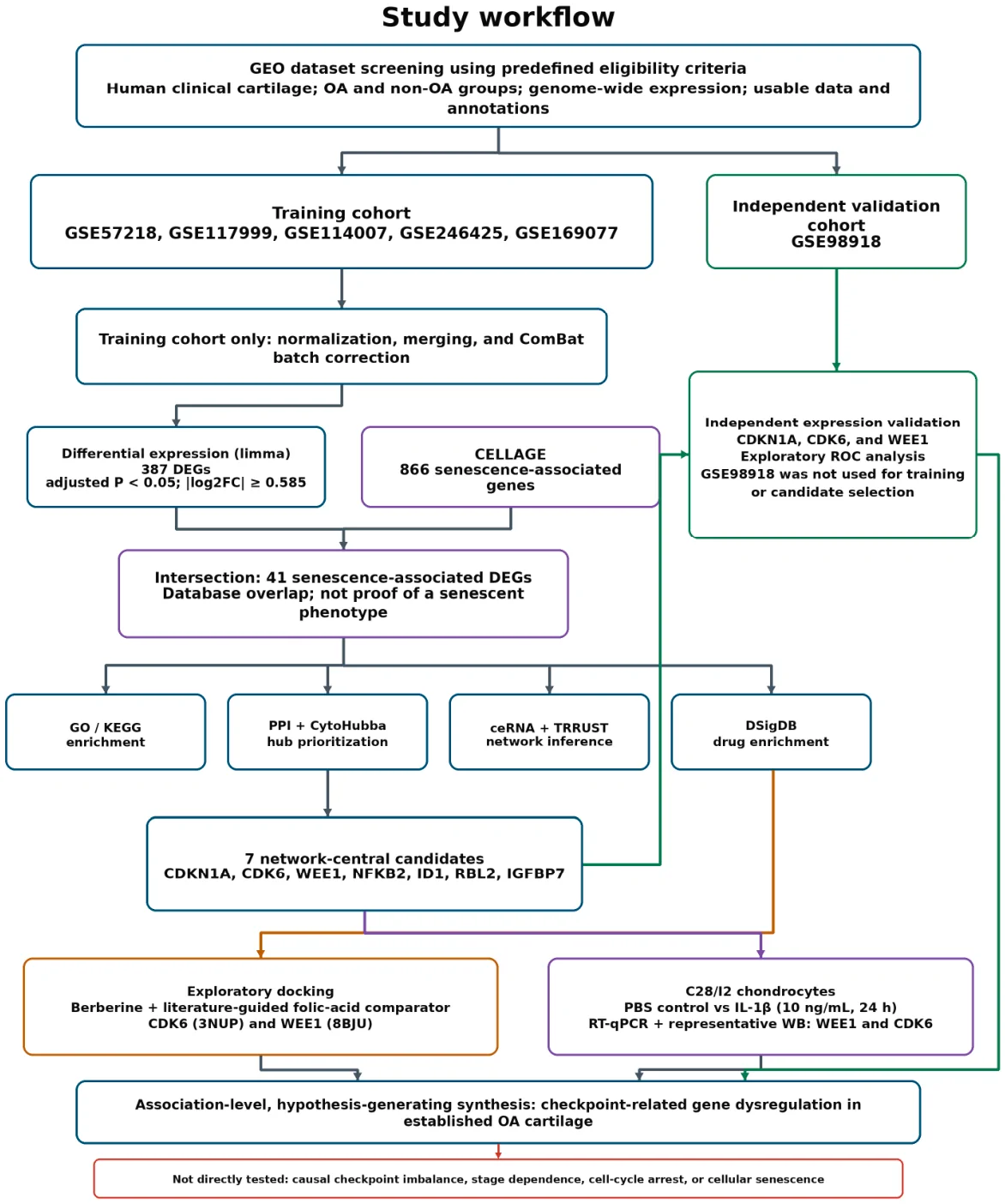
Study workflow. Five GEO datasets (GSE57218, GSE117999, GSE114007, GSE246425, and GSE169077) were integrated as the training cohort; GSE98918, which contains meniscal tissue, was reserved exclusively for independent cross-tissue validation. Only the training datasets underwent merging, ComBat batch correction, differential expression analysis, and candidate prioritization. The 387 OA-associated DEGs were intersected with 866 CELLAGE genes, yielding 41 senescence-associated DEGs, a label denoting database overlap rather than proof of a senescent phenotype. CDKN1A, CDK6, and WEE1 were evaluated in GSE98918, and WEE1 and CDK6 were examined in IL-1β-treated C28/I2 cells. The final synthesis is association-level and hypothesis-generating; causal checkpoint imbalance, stage dependence, cell-cycle arrest, and cellular senescence were not directly tested.

The study workflow is shown in Figure 1.

### 2.1. Data Acquisition and Preprocessing

#### 2.1.1. GEO Dataset Selection

Gene-expression datasets were retrieved from GEO [23,24]. For the training analysis, inclusion required: (1) human knee articular-cartilage tissue or primary cartilage-derived chondrocyte profiles with defined OA and non-OA/healthy comparison groups; (2) genome-wide expression profiling; (3) an accessible raw or normalized expression matrix; and (4) sample annotations adequate to define the comparison groups. Exclusion criteria were: (1) animal studies; (2) unrelated cell-line-only datasets; (3) duplicated or overlapping cohorts; (4) unavailable or unusable expression matrices; and (5) insufficient grouping information. Because GSE98918 contains meniscal rather than articular-cartilage tissue, it was not merged with the training data and was used only as an independent cross-tissue validation cohort.

GSE57218, GSE117999, GSE114007, GSE246425, and GSE169077 were used as the training cohort. GSE98918 was pre-specified as an independent cross-tissue validation cohort and was not included in training, merging, batch correction, hub-gene selection, or model development. The characteristics and analytical roles of all GEO datasets are summarized in Table 1.

#### 2.1.2. Normalization and Batch Correction

Microarray data were log2-transformed when necessary and normalized using the limma package (version 3.62.2; Bioconductor, https://bioconductor.org/packages/limma/, acceessed on 1 January 2026) [25]. Probe identifiers were mapped to gene symbols, and multiple probes assigned to the same symbol were averaged. Only the five training datasets were merged. Batch effects were adjusted using ComBat implemented in the sva package (version 3.50.0; Bioconductor, https://bioconductor.org/packages/sva/, acceessed on 1 January 2026) [26]. Principal-component analysis (PCA) was used to inspect dataset-driven structure before and after correction; PC1 and PC2 denote the first and second principal components.

### 2.2. Senescence-Associated Differential Expression

#### 2.2.1. Differential Expression

Differential expression in the integrated training cohort was analyzed with the limma package (version 3.62.2; Bioconductor, https://bioconductor.org/packages/limma/, acceessed on 1 January 2026) using linear modeling (lmFit), empirical-Bayes moderation (eBayes), and Benjamini–Hochberg adjustment. [25]. Differentially expressed genes (DEGs) were defined as genes with adjusted *p* < 0.05 and |log2 fold change| ≥ 0.585.

#### 2.2.2. Intersection with CELLAGE

The OA-associated DEGs were intersected with 866 senescence-associated genes compiled from the CELLAGE resource [27,28]. The resulting genes are referred to as senescence-associated DEGs; this label denotes database overlap and does not prove a senescent phenotype.

### 2.3. Gene Ontology and Pathway Enrichment Analysis

Gene Ontology (GO) and Kyoto Encyclopedia of Genes and Genomes (KEGG) enrichment analyses were performed using the clusterProfiler package (version 4.10.0; Bioconductor, https://bioconductor.org/packages/clusterProfiler/, acceessed on 1 January 2026). [29,30,31]. To account for multiple testing, *p* values were adjusted using the Benjamini–Hochberg method. Terms with an adjusted *p* value < 0.05 were considered statistically significant. Enrichment results were interpreted at the pathway-association level and not as direct evidence of pathway activation.

### 2.4. Network Analyses

#### 2.4.1. PPI and Hub-Gene Prioritization

A protein–protein interaction (PPI) network was generated using the STRING database (version 12.0; https://string-db.org/, acceessed on 1 January 2026) at a minimum interaction score of 0.400 and visualized using Cytoscape software (version 3.10.1; https://cytoscape.org/, acceessed on 1 January 2026) [32,33]. The 41 senescence-associated DEGs were used as the STRING input set, with no additional external interactors. The cytoHubba plugin (version 0.1; https://apps.cytoscape.org/apps/cytohubba, acceessed on 1 January 2026) was applied using MCC, DMNC, MNC, Degree, BottleNeck, EcCentricity, Closeness, Radiality, Betweenness, and Stress algorithms. [34]. Genes repeatedly ranked near the top across these algorithms were intersected to prioritize seven network-central candidates. Network centrality was not interpreted as causality.

#### 2.4.2. ceRNA and Transcription-Factor Networks

The competing endogenous RNA (ceRNA) network was assembled from miRNA-target predictions obtained from miRanda (version 3.3a; http://www.microrna.org/, acceessed on 1 January 2026), miRDB (version 6.0; https://mirdb.org/, acceessed on 1 January 2026), miRTarBase (version 9.0; https://mirtarbase.cuhk.edu.cn/, acceessed on 1 January 2026), and TargetScanHuman (release 8.0; https://www.targetscan.org/, acceessed on 1 January 2026). Only interactions supported by all four sources were retained [35,36,37].Functional association analysis was performed using GeneMANIA (version 3.5.3; https://genemania.org/, acceessed on 1 January 2026). For a readable main figure, up to two highest-degree miRNAs linked to each of CDKN1A, CDK6, WEE1, and RBL2 were first retained, additional miRNAs were added by total degree to a maximum of 12, and the 18 highest-degree-connected lncRNAs were displayed. The complete predicted network is provided as Supplementary Figure S1.Transcription-factor (TF) relationships were obtained from the TRRUST database (version 2; https://www.grnpedia.org/trrust/, acceessed on 1 January 2026) and examined in GSE98918 [38,39].

### 2.5. Drug Enrichment and Molecular Docking

#### 2.5.1. Drug-Enrichment Analysis and Candidate Selection

DSigDB drug-signature enrichment was performed using the clusterProfiler package (version 4.10.0; Bioconductor, https://bioconductor.org/packages/clusterProfiler/, acceessed on 1 January 2026) based on the Drug Signatures Database (DSigDB, version 1.0; https://dsigdb.tanlab.org/DSigDBv1.0/, acceessed on 1 January 2026) [40]. Terms with nominal *p* < 0.05 and adjusted *p* < 0.05 were considered enrichment signals. Berberine was selected for docking because it appeared among the enrichment results and has experimental literature relevant to OA [41,42,43]. Folic acid was not among the visually prominent top-ranked enrichment terms; it was included post hoc as a literature-guided comparator because observational studies have linked folate deficiency to knee-OA severity and folate-derived materials have been explored in OA-related models [44,45,46]. Thus, folic acid should not be described as an enrichment-prioritized therapeutic candidate.

#### 2.5.2. Molecular Docking

Crystal structures of CDK6 (PDB ID: 3NUP) and WEE1 (PDB ID: 8BJU) were obtained from the RCSB Protein Data Bank. The three-dimensional structures of berberine and folic acid were obtained from the PubChem database. Molecular docking was performed using the CB-Dock2 web server (https://cadd.labshare.cn/cb-dock2/, acceessed on 1 January 2026), and Vina-derived affinity scores (kcal/mol) were used for descriptive comparison [47]. Docking results provide only computational structural plausibility; no biochemical binding, target engagement, cellular activity, or therapeutic efficacy was experimentally validated.

### 2.6. Cell-Culture Evaluation

#### 2.6.1. Cell Culture and IL-1β Treatment

The C28/I2 human chondrocyte cell line was purchased from Sigma-Aldrich(SCC043; St. Louis, MO, USA) and cultured in Dulbecco’s modified Eagle’s medium (DMEM)/F-12 (Gibco, Thermo Fisher Scientific; Waltham, MA, USA) supplemented with 10% fetal bovine serum ((Sigma-Aldrich, 12103C; St. Louis, MO, USA)) and 1% penicillin/streptomycin (Gibco, Thermo Fisher Scientific; Waltham, MA, USA). Cells were treated with recombinant human IL-1β (MedChemExpress, HY-P73149; Monmouth Junction, NJ, USA) at 10 ng/mL for 24 h; cells treated with phosphate-buffered saline (PBS; Servicebio, G4202-500ML) served as controls. This acute exposure was considered an OA-like inflammatory chondrocyte model rather than a validated cellular senescence model [48,49].

#### 2.6.2. RT-qPCR

Total RNA was extracted from C28/I2 cells using TRIzol reagent (Invitrogen, 15596018CN; Carlsbad, CA, USA). cDNA was prepared using the Evo M-MLV Reverse Transcription Kit (Accurate Biology, AG11728; Changsha, China) in a T100 Thermal Cycler (Bio-Rad, 1861096; Hercules, CA, USA). Genomic DNA was removed at 42 °C for 2 min when required, followed by reverse transcription at 37 °C for 15 min, and enzyme inactivation at 85 °C for 5 s. qPCR was performed using SYBR Green Pro Taq HS qPCR Mix (Accurate Biology, AG11702; Changsha, China) on a QuantStudio 3 Real-Time PCR System (Applied Biosystems, A28567; Foster City, CA, USA) in a final reaction volume of 20 μL containing SYBR Green master mix, forward and reverse primers, cDNA template, and nuclease-free water. Cycling comprised initial denaturation at 95 °C for 30 s, followed by 40 cycles of 95 °C for 5 s and 60 °C for 30 s, and subsequent melting-curve analysis. Primer sequences are listed in Supplementary Table S1. Expression was normalized to β-ACTIN and calculated using the 2^−ΔΔCt^ method.

#### 2.6.3. Western Blotting

Total cellular protein was extracted using RIPA lysis buffer (Beyotime, P0013B; Shanghai, China) supplemented with protease inhibitor cocktail (MedChemExpress, HY-K0010; Monmouth Junction, NJ, USA). Equal amounts of protein (20 μg per lane) were separated on 10% SDS-PAGE gels (Epizyme, PG112; Shanghai, China) and transferred to 0.45 μm PVDF membranes (Millipore, IPVH00010; Burlington, MA, USA). After blocking with non-fat milk powder (Servicebio, GC310001-100G; Wuhan, China), membranes were incubated overnight at 4 °C with anti-WEE1 (Cell Signaling Technology, #13084, 1:1000; Danvers, MA, USA), anti-CDK6 (Proteintech, 14052-1-AP, 1:1000; Rosemont, IL, USA), or anti-β-actin (Proteintech, 20536-1-AP, 1:5000), followed by HRP-conjugated secondary antibody (Proteintech, RGAR301, 1:5000). Signals were developed with ECL substrate (Biosharp, BL520A; Hefei, China) and imaged using a ChemiScope 6000 system (CLINX, Shanghai, China). Western blotting was performed in three independent biological experiments. Representative blots are shown, and the source images underlying the displayed representative panels are provided in Supplementary Figure S2. The immunoblot findings are presented qualitatively; densitometric quantification and protein-level inferential statistics were not performed.

### 2.7. Statistical Analysis

Bioinformatic analyses were conducted using R software (version 4.4.1). Differential expression analysis, batch correction, enrichment analysis, and ROC analysis were performed using the corresponding R packages described above. ROC curves in GSE98918 were generated using the pROC package (version 1.18.5; CRAN, https://cran.r-project.org/package=pROC, acceessed on 1 January 2026). limma was used for differential expression, sva for ComBat correction, clusterProfiler for enrichment, and Cytoscape 3.10.1 for networks. ROC curves in GSE98918 were generated with pROC; the area under the curve (AUC) and displayed 95% confidence intervals were calculated within that cross-tissue validation dataset. These ROC analyses were exploratory and were not used to claim clinical diagnostic validity. RT-qPCR results are shown as mean ± SD from three independent biological replicates and were compared by an unpaired two-tailed Student’s t test in GraphPad Prism 9.0. *p* < 0.05 was considered significant. Western blot findings are presented as representative qualitative results and were not included in protein-level inferential statistical analysis.

## 3. Results

### 3.1. Integrated Transcriptomic Analysis

#### 3.1.1. Normalization, Batch Correction, and DEGs

**Figure 2:**
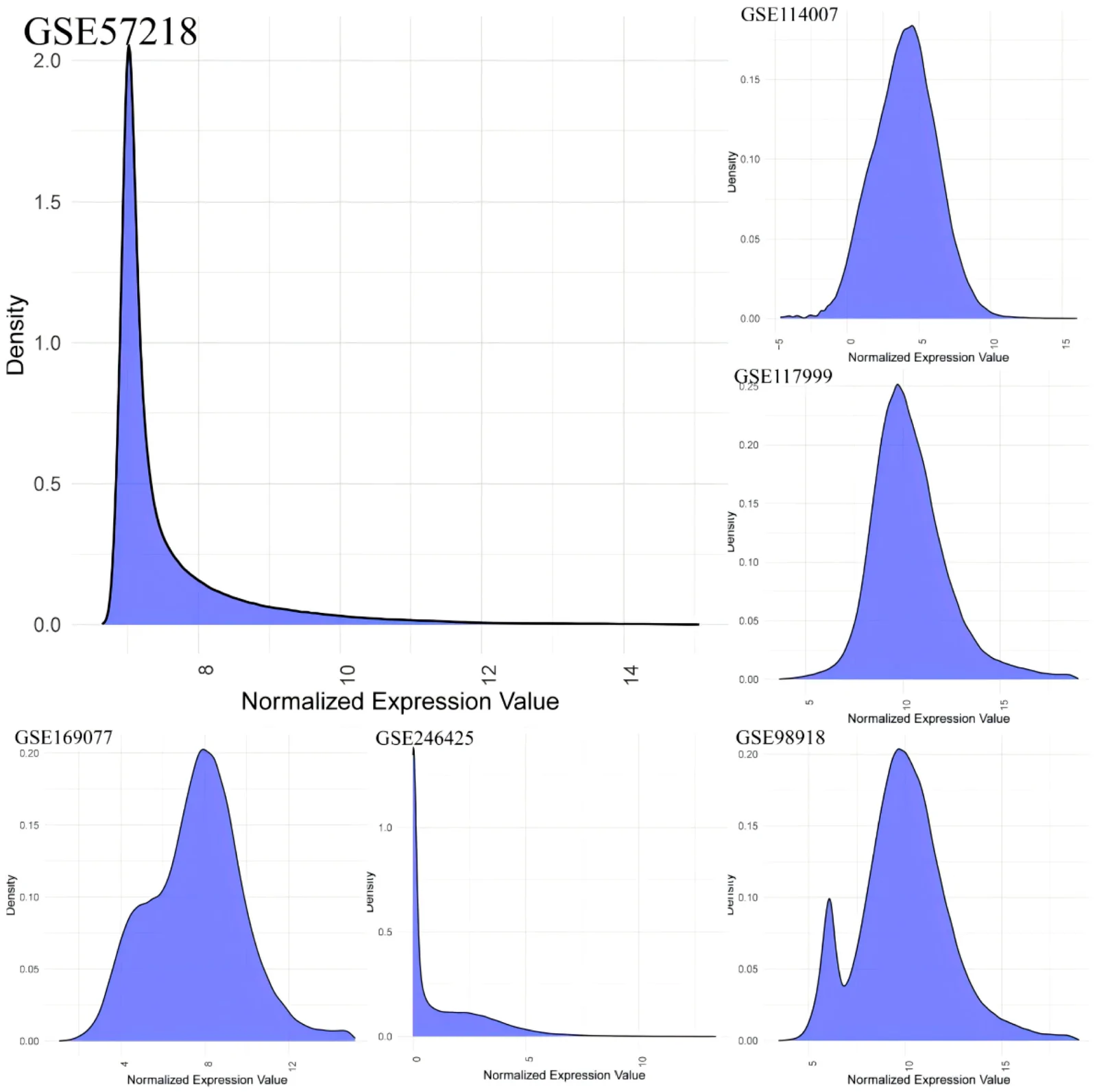
Density plots of normalized expression values. GSE57218, GSE117999, GSE114007, GSE246425, and GSE169077 constitute the training cohort; GSE98918 is displayed separately as an independent cross-tissue validation cohort and is not merged into the training analysis. The *x*-axis is normalized expression and the *y*-axis is density.

**Figure 3:**
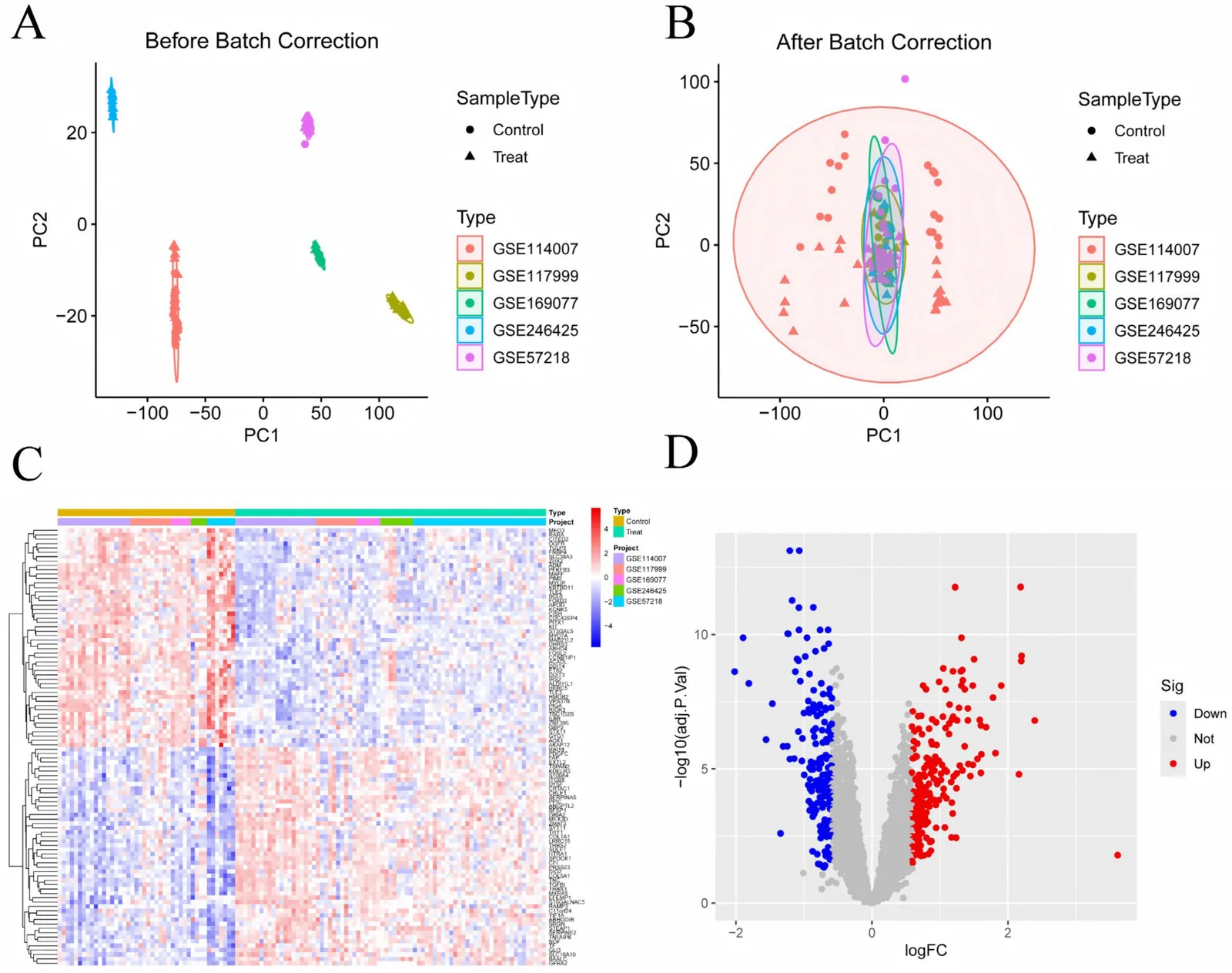
Training cohort integration and differential expression. (**A**) PCA before and (**B**) after ComBat batch correction. PC1 and PC2 are the first and second principal components. Circles indicate non-OA control cartilage; triangles indicate OA cartilage. Colors identify GEO datasets. (**C**) Heatmap of the integrated training cohort after correction; color denotes relative standardized expression. (**D**) Volcano plot; red, blue, and gray indicate upregulated, downregulated, and non-significant genes, respectively.

The five training datasets were normalized, merged, and batch-corrected. The normalized expression distributions are shown for the five training datasets and, separately, for the independent cross-tissue GSE98918 cohort (Figure 2). Before ComBat adjustment, PCA showed substantial dataset-driven separation; after adjustment, the training samples showed improved overlap (Figure 3A,B). limma identified 387 DEGs at adjusted *p* < 0.05 and |log2 fold change| ≥ 0.585 (Figure 3C,D).

#### 3.1.2. CELLAGE Intersection

**Figure 4:**
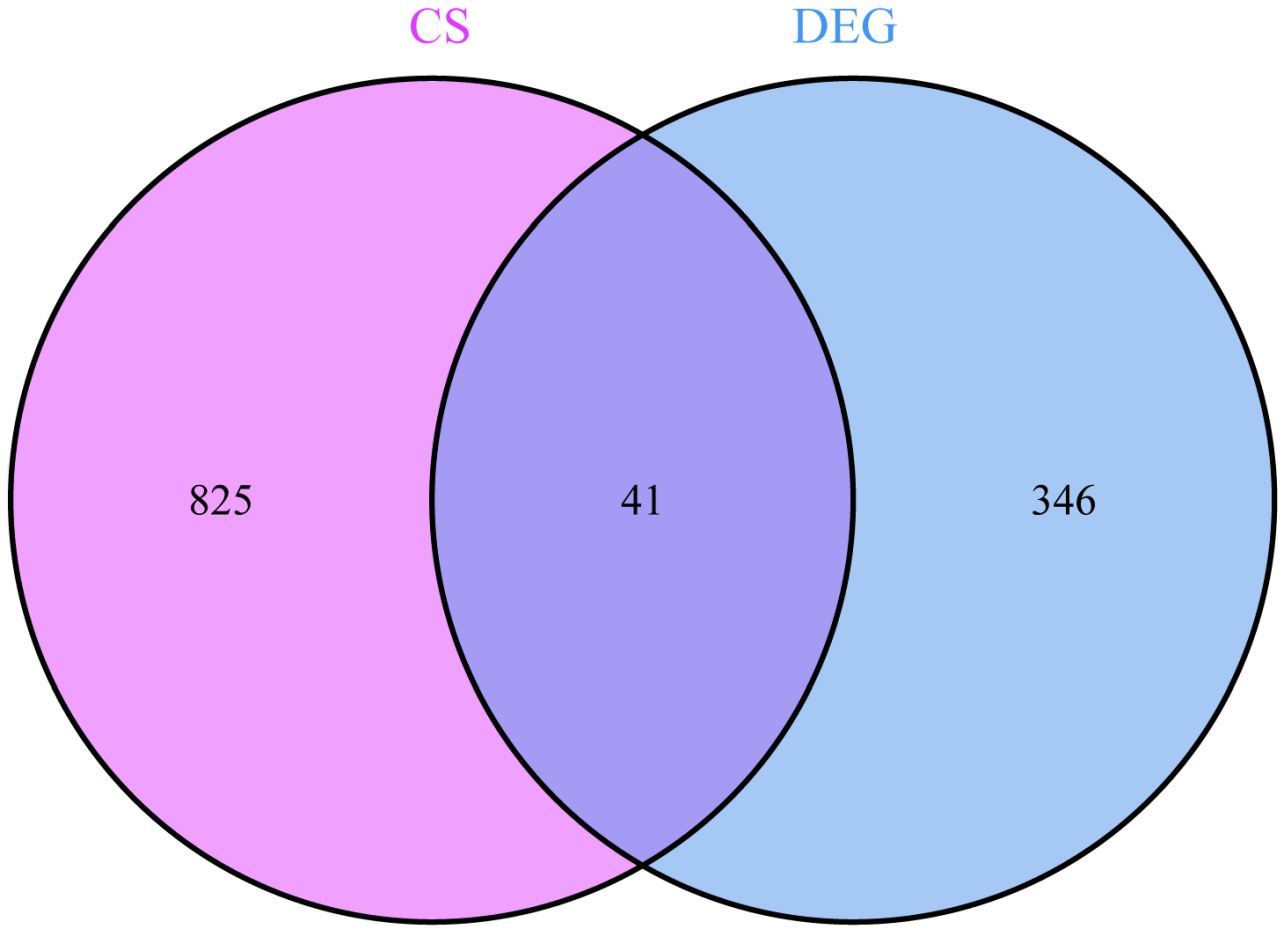
Intersection of CELLAGE genes (CS) and OA-associated differentially expressed genes (DEGs). Forty-one genes were shared.

The intersection of 387 OA-associated DEGs with 866 CELLAGE genes yielded 41 senescence-associated DEGs (Figure 4). This overlap identifies genes annotated in both sets but does not demonstrate senescence in the studied samples.

### 3.2. Functional Enrichment

**Figure 5:**
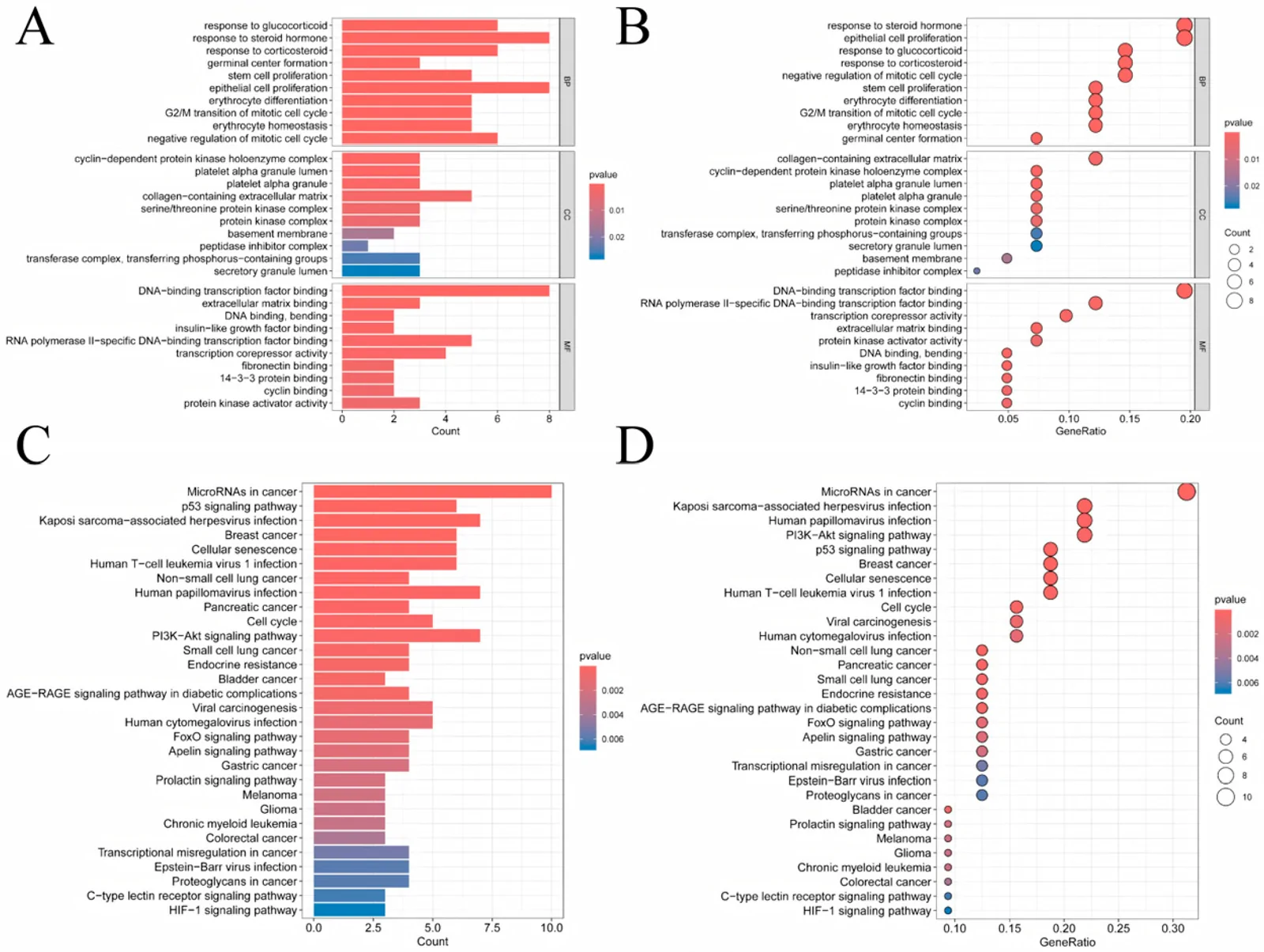
GO and KEGG enrichment of the 41 senescence-associated DEGs. (**A**,**B**) Representative GO terms. (**C**,**D**) Representative KEGG pathways. Bar length and dot size indicate gene count or ratio as labeled, and color gradients indicate enrichment significance.

After multiple-testing correction, the 41-gene set showed enrichment in cell-cycle-associated biological processes, with G2/M transition and negative regulation of the mitotic cell cycle among the leading annotations. Collagen-containing extracellular matrix and transcription-factor-binding terms were also represented. KEGG analysis included cellular senescence, p53 signaling, and microRNA-related pathways among the reported annotations (Figure 5). These findings indicate over-representation of annotated pathways and do not establish pathway activation, p53 activity, or checkpoint function.

### 3.3. PPI Network and Hub-Gene Prioritization

**Figure 6:**
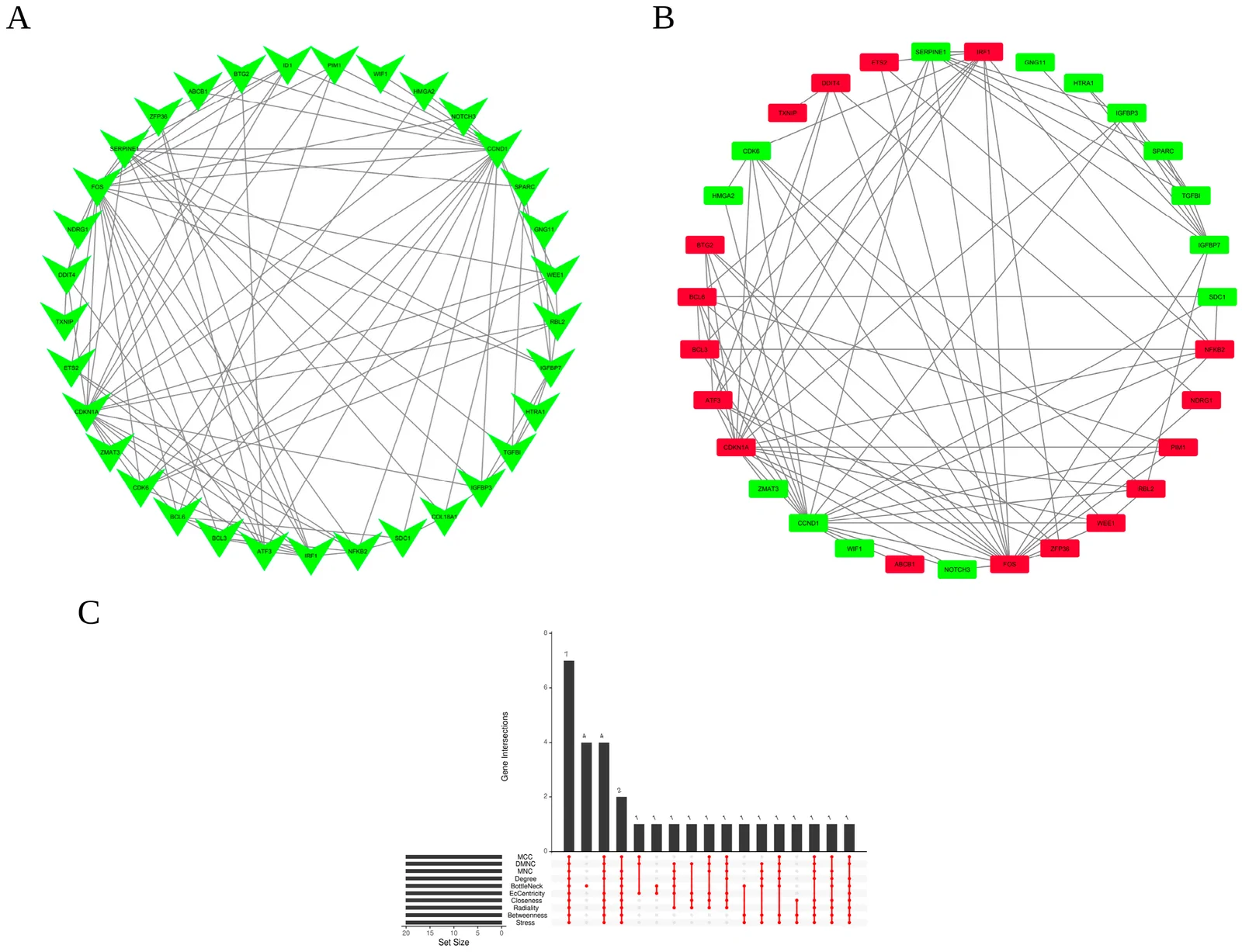
PPI network and hub-gene prioritization. (**A**) STRING protein–protein interaction network constructed from the 41 senescence-associated differentially expressed genes (DEGs). No external interactors were added. (**B**) Expression-direction mapping of the connected interaction network. Red nodes indicate genes upregulated in OA cartilage, whereas green nodes indicate genes downregulated in OA cartilage. (**C**) UpSet plot showing the intersection of hub-gene rankings obtained from ten CytoHubba algorithms. Red dots indicate algorithms included in a specific intersection, whereas gray dots indicate algorithms not included in that intersection. Vertical bars represent the number of genes shared among the corresponding algorithm combinations.

The PPI network generated from the 41 senescence-associated DEGs, with no additional external interactors, contained 41 nodes and 90 edges (Figure 6A). Differential-expression mapping showed both upregulated and downregulated genes within the connected interaction network (Figure 6B). Across ten CytoHubba centrality algorithms, the intersection of repeatedly highly ranked genes yielded CDKN1A, CDK6, WEE1, NFKB2, ID1, RBL2 and IGFBP7 (Figure 6C). These genes were carried forward as network-central candidates rather than causal drivers.

### 3.4. Regulatory Networks

**Figure 7:**
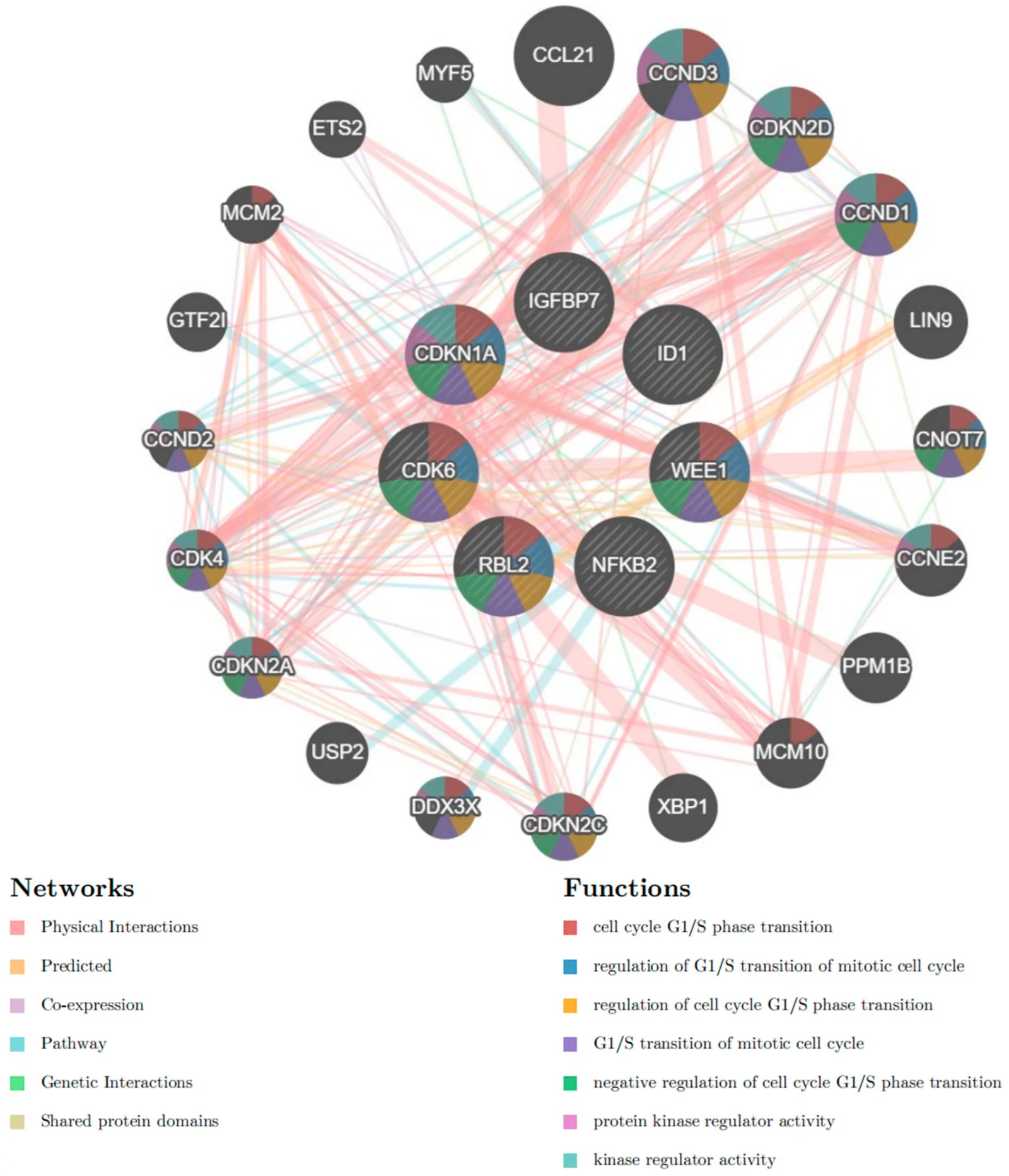
GeneMANIA functional-association network. Edge colors distinguish physical interaction, prediction, co-expression, pathways, genetic interactions, and shared-domain evidence. Colored sectors within nodes identify the enriched functional categories shown in the legend.

GeneMANIA connected the seven candidates to genes annotated for G1/S regulation, cell-cycle transition, and related functions (Figure 7). The edge and node colors identify the source of functional association and enriched functional category, respectively.

**Figure 8:**
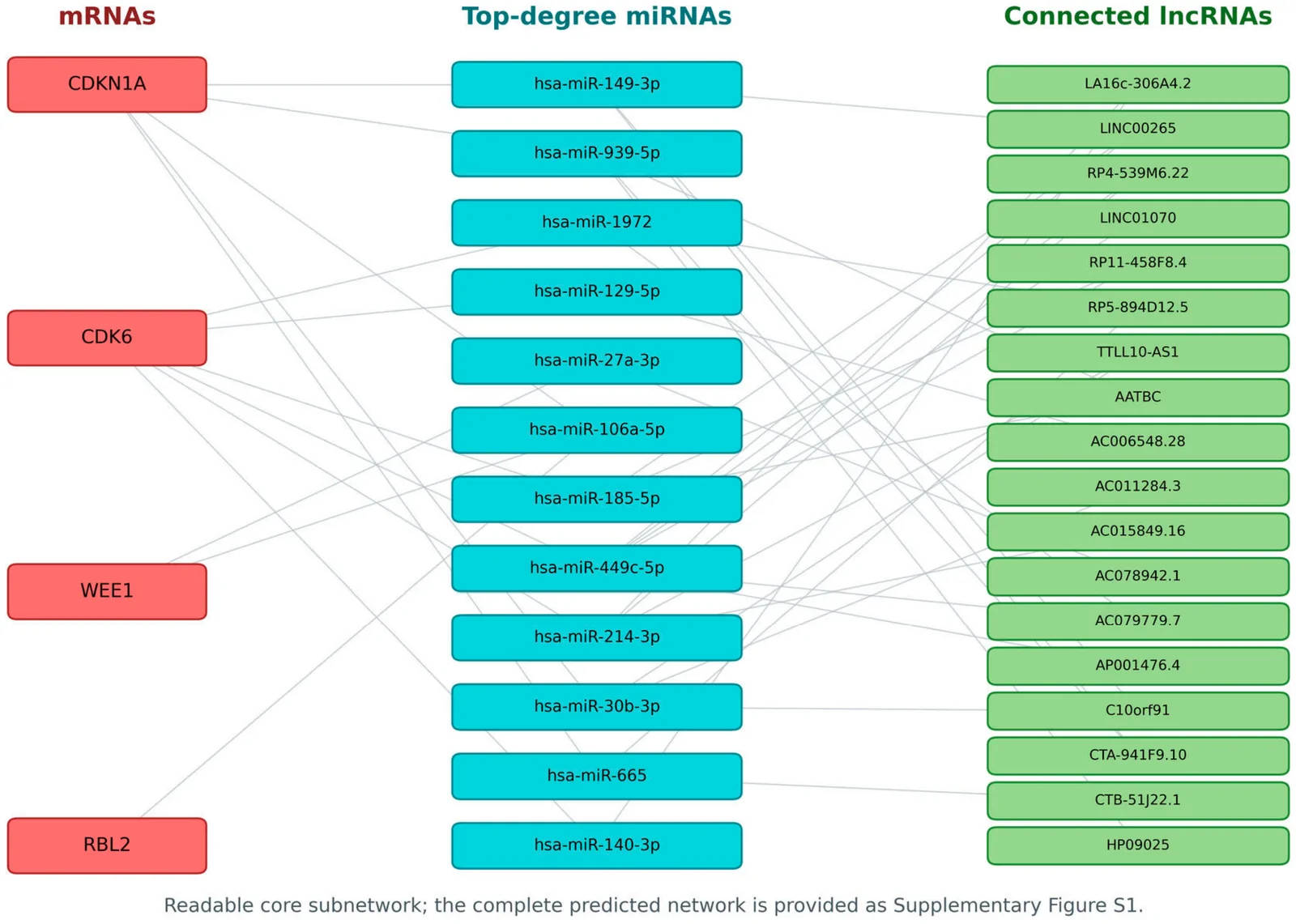
Readable core ceRNA subnetwork. Red nodes are mRNAs, cyan nodes are miRNAs, and green nodes are lncRNAs. Up to two highest-degree miRNAs per mRNA were retained first, the list was extended to 12 miRNAs by total degree, and the 18 highest-degree, connected lncRNAs were displayed. Lines denote predicted interactions. The complete network is in Figure S1.

A predicted ceRNA network was obtained for CDKN1A, CDK6, WEE1, and RBL2. Figure 8 shows a degree-based core subnetwork so that labels remain legible; the complete network is provided as Figure S1. These edges represent database-supported predictions and were not experimentally validated.

**Figure 9:**
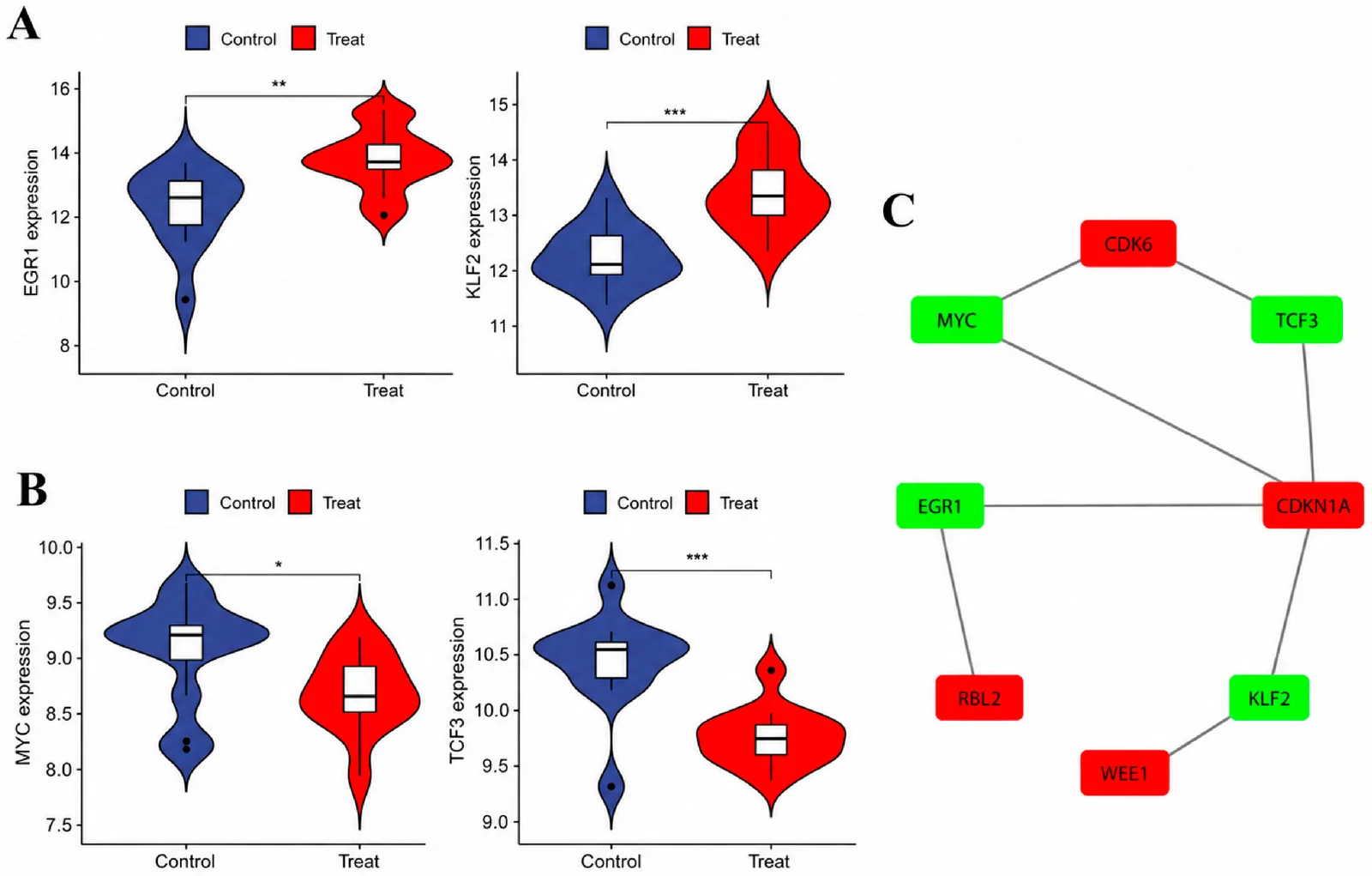
TF expression and regulatory networks. (**A**,**B**) GSE98918 meniscus expression distributions; Control denotes non-OA meniscal tissue and Treat denotes OA meniscal tissue. * *p* < 0.05, ** *p* < 0.01, *** *p* < 0.001. (**C**) Green nodes are TFs and red nodes are hub genes; edges are TRRUST-curated regulatory relationships.

TRRUST identified EGR1, KLF2, MYC, and TCF3 as candidate upstream regulators with expression differences in the independent GSE98918 meniscus cohort (Figure 9A,B). The network illustrates curated regulatory relationships to the selected hub genes (Figure 9C) but does not demonstrate regulatory activity in OA cartilage or meniscus.

### 3.5. Exploratory Drug Enrichment and Docking

**Figure 10:**
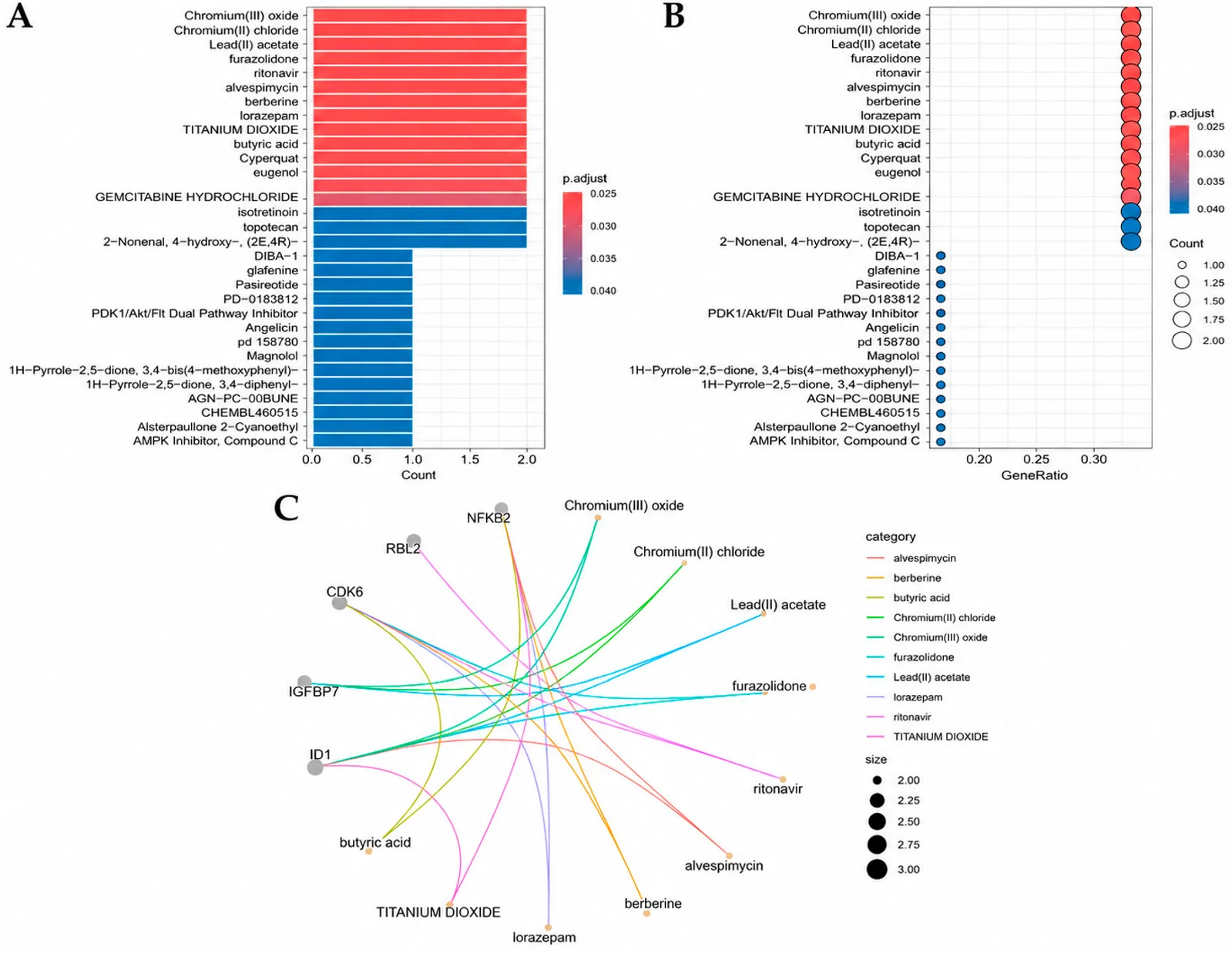
DSigDB enrichment. (**A**) Bar plot and (**B**) bubble plot of displayed enriched compound signatures. Color encodes the displayed adjusted *p*-value scale; bar length and dot size encode count or gene ratio as labeled. (**C**) Compound-gene network. Gray nodes are hub genes and colored nodes are enrichment-derived compounds. Folic acid is not shown as a top enrichment-derived node.

DSigDB analysis yielded multiple compound-associated signatures, including a berberine-related signal (Figure 10). Folic acid was not among the visually prominent top terms in the displayed enrichment result and was therefore treated as a post hoc, literature-guided comparator, not as a top enrichment-derived candidate.

**Figure 11:**
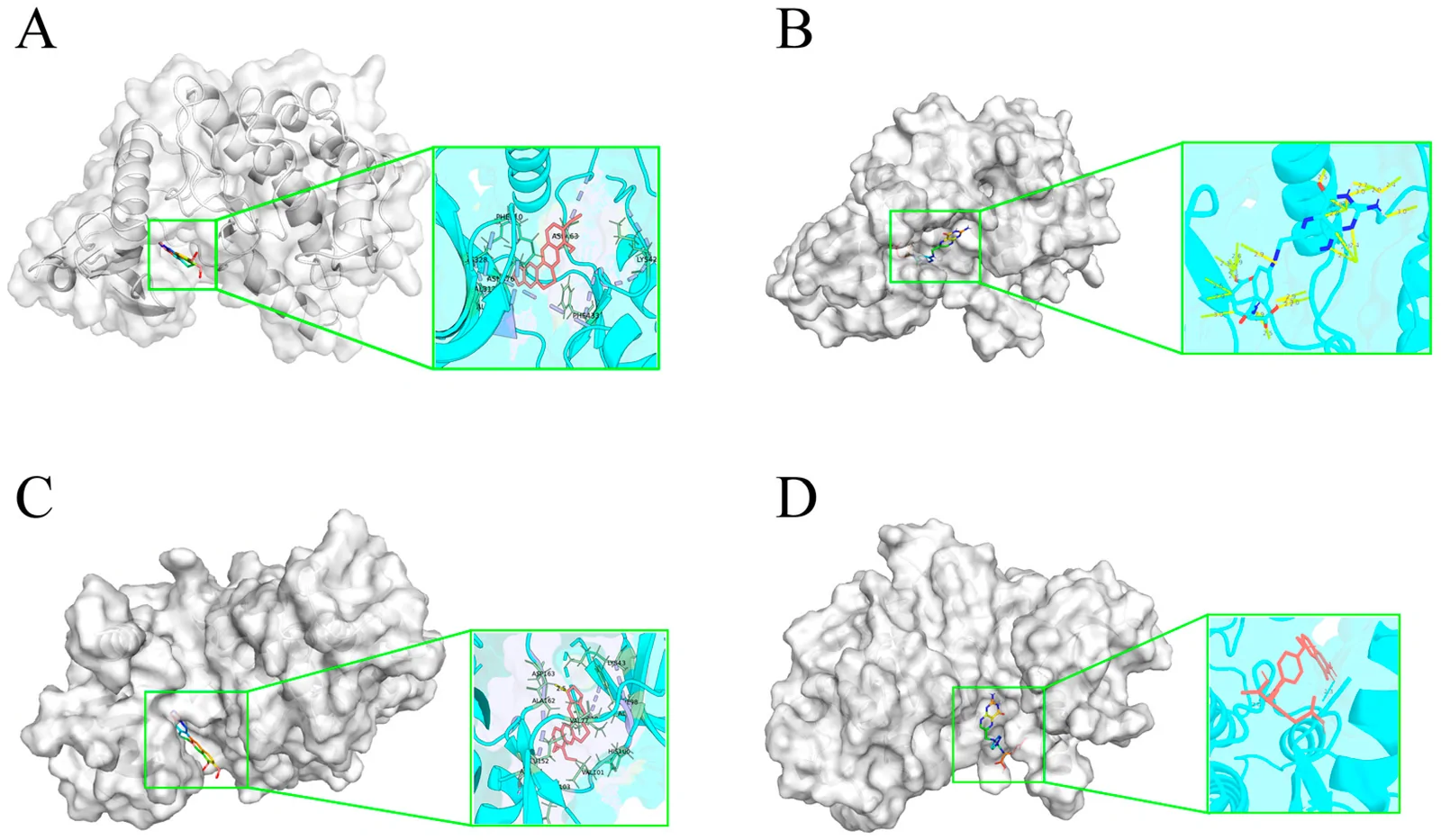
Exploratory molecular docking. (**A**) Berberine-WEE1 (PDB: 8BJU). (**B**) Folic acid-WEE1 (PDB: 8BJU). (**C**) Berberine-CDK6 (PDB: 3NUP). (**D**) Folic acid-CDK6 (PDB: 3NUP). Insets display the predicted binding pockets. Docking does not establish target engagement or efficacy.

Docking scores were −7.1 kcal/mol for berberine-WEE1, −9.5 kcal/mol for folic acid-WEE1, −8.6 kcal/mol for berberine-CDK6, and −7.1 kcal/mol for folic acid-CDK6 for the best-ranked poses (Figure 11; Table 2, Table 3, Table 4 and Table 5). The folic acid-WEE1 score was the most negative of the four comparisons, indicating only a more favorable predicted pose under this docking procedure. It does not demonstrate binding or biological modulation of WEE1.

### 3.6. Independent Cross-Tissue Validation in GSE98918

**Figure 12:**
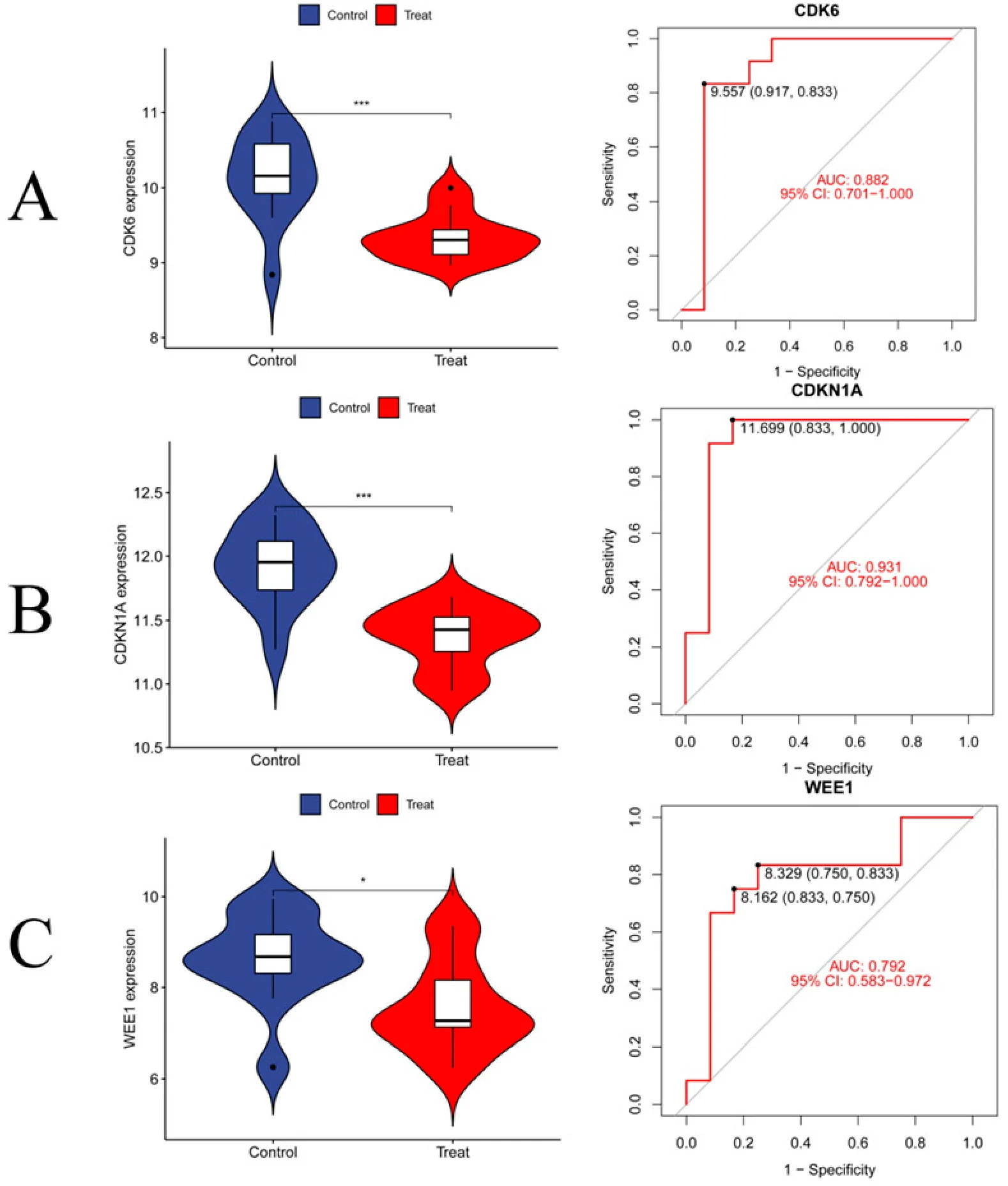
Independent cross-tissue validation in GSE98918. Expression distributions and exploratory ROC curves are shown for (**A**) CDK6, (**B**) CDKN1A, and (**C**) WEE1. Control denotes non-OA meniscal tissue, and Treat denotes OA meniscal tissue. AUCs are within-dataset descriptive estimates and should not be interpreted as validated diagnostic performance. Statistical significance was determined using the indicated statistical test. * *p* < 0.05; *** *p* < 0.001.

CDK6, CDKN1A, and WEE1 expression was lower in OA than in non-OA meniscal tissue in GSE98918 (Figure 12). Exploratory within-cohort AUCs were 0.882 for CDK6, 0.931 for CDKN1A, and 0.792 for WEE1. Because GSE98918 is a meniscus dataset rather than a cartilage dataset, these analyses are treated as cross-tissue validation of expression direction and separation only; they do not establish cartilage-specific diagnostic biomarkers, clinical thresholds, or generalizable performance.

### 3.7. RT-qPCR and Representative Immunoblot Evaluation

**Figure 13:**
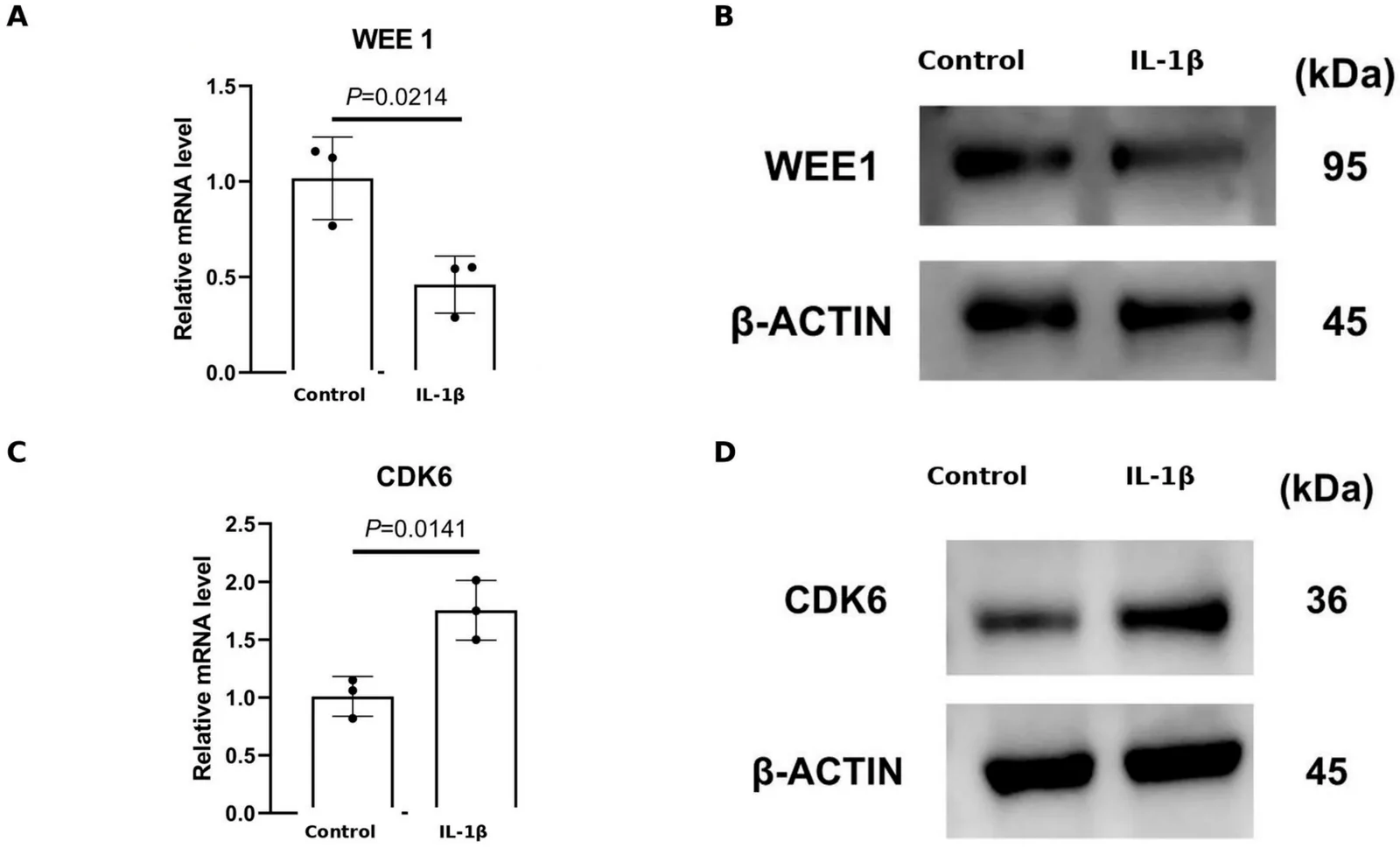
Evaluation of WEE1 and CDK6 in human C28/I2 chondrocytes following treatment with 10 ng/mL IL-1β for 24 h. (**A**) RT-qPCR analysis of WEE1 mRNA. (**B**) Representative WEE1 and β-actin immunoblots. (**C**) RT-qPCR analysis of CDK6 mRNA. (**D**) Representative CDK6 and β-actin immunoblots. PBS-treated cells served as controls. RT-qPCR data are presented as the mean ± SD from three independent biological replicates. Western blotting was performed in three independent biological experiments; representative blots are shown. The source image underlying the displayed panels is provided in Supplementary Figure S2. The immunoblot findings are presented qualitatively without densitometric or protein-level inferential statistics.

In IL-1β-treated C28/I2 cells, WEE1 mRNA was lower than in PBS-treated controls (*p* = 0.0214), whereas CDK6 mRNA was higher (*p* = 0.0141) (Figure 13A,C). Western blotting was performed in three independent biological experiments. Representative blots qualitatively showed weaker WEE1 and stronger CDK6 bands relative to β-actin (Figure 13B,D), and the corresponding source image is provided in Supplementary Figure S2. These immunoblot observations are descriptive and are not used for quantitative protein-level inference.

The CDK6 direction in this acute inflammatory cell model differed from the reduction observed in clinical cartilage datasets. This discordance indicates model dependence or biological heterogeneity; it does not demonstrate an early-to-late transition. The experiment also did not assess cell-cycle distribution, p53 activity, senescence markers, or SASP factors.

## 4. Discussion

### 4.1. Senescence-Associated Checkpoint Signals in Established OA

This integrated analysis identified 41 OA-associated DEGs that overlapped CELLAGE annotations and prioritized seven network-central genes. The most reproducible directional finding was lower WEE1 expression in OA cartilage and lower WEE1 mRNA in an IL-1β-treated human chondrocyte model. CDK6, by contrast, was lower in OA cartilage datasets but higher after acute IL-1β–exposure. These observations support further study of checkpoint-related genes in established OA but do not establish a checkpoint-imbalance mechanism.

CDKN1A is a well-known p21-encoding mediator of stress-induced cell-cycle arrest, and WEE1 regulates G2/M checkpoint timing [17,18,19,20]. However, CDKN1A was reduced in the independent cross-tissue validation cohort. Consequently, the present data do not support a conclusion of p53-mediated CDKN1A activation. Enrichment of a p53-signaling gene set is an association at the pathway level and cannot substitute for p53 activation assays or downstream functional measurements.

The remaining prioritized genes also have plausible links to senescence-associated secretion or cell-cycle regulation: IGFBP7 has been described as a senescence-associated secretory component, ID proteins participate in chondrocyte cell division control, and RBL2-related signaling contributes to cell-cycle arrest [50,51,52]. These literature links support prioritization but do not validate the inferred network in OA.

### 4.2. CDK6 Discordance Is Not Evidence of Stage Dependence

The opposite CDK6 directions in clinical cartilage and cultured chondrocytes can arise from several non-exclusive sources: cell-type composition in bulk tissue, differences in patient phenotype and OA severity, acute versus chronic exposure, immortalized cell behavior, culture conditions, and post-transcriptional regulation. Clinical OA tissue used in many GEO cohorts is obtained during joint replacement and therefore mainly represents established disease. The 24 h IL-1β model captures an acute inflammatory perturbation and cannot be assigned to a defined early OA stage.

The discordant CDK6 pattern should therefore not be interpreted as evidence of a compensatory early upregulation followed by late exhaustion. A time course, matched early- and late-stage clinical tissue and quantitative mRNA/protein measurements would be needed to test stage-related regulation. FACS-based cell-cycle analysis would additionally be required to determine whether CDK6 differences correspond to altered G1/S progression.

### 4.3. WEE1 as a Candidate, Not Proof of G2/M Failure

WEE1 reduction is compatible with altered checkpoint-related biology, but expression alone does not measure kinase activity, inhibitory CDK phosphorylation, DNA-damage signaling, or G2/M distribution [19,20]. The qPCR result provides quantitative support for reduced WEE1 transcript after IL-1β exposure, while the representative immunoblot evaluation remains qualitative. Quantitative protein-level validation would require replicate-level densitometric analysis, together with functional perturbation and cell-cycle assays, before a causal role in senescence or checkpoint failure can be attributed.

### 4.4. Exploratory Compound Signals

Berberine has shown anti-inflammatory and cartilage-protective effects in experimental OA contexts [41,42,43]. Folate status has been associated with radiographic or clinical knee-OA severity in observational studies [45,46], and folate-derived carbon dots have been explored experimentally [44]. These publications justify discussion of biological relevance but do not validate direct modulation of CDK6 or WEE1.

The more negative folic acid–WEE1 docking score indicates only a preferred computational pose within the selected structures and algorithm. It cannot be interpreted as binding, restoration of WEE1 function, or therapeutic efficacy. In particular, folic acid was a literature-guided comparator rather than a top displayed DSigDB hit. The compound analyses should therefore be regarded as hypothesis-generating.

### 4.5. Cautious Working Framework

**Figure 14:**
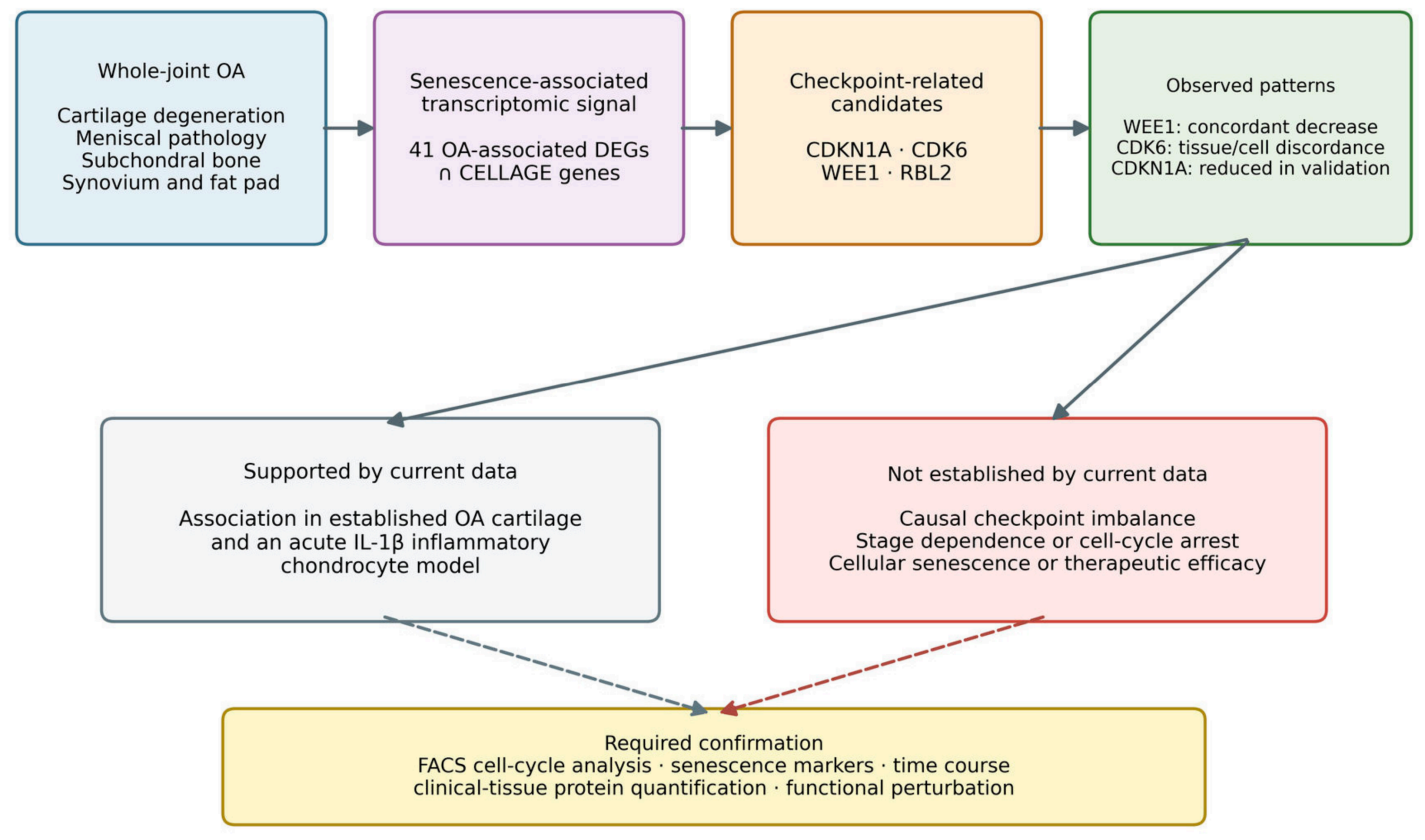
Hypothesis-generating framework. Whole-joint OA provides the clinical context; transcriptomic analyses identify senescence-associated and checkpoint-related candidates. The observed WEE1, CDK6, and CDKN1A patterns support association-level interpretations only. Solid arrows indicate the analytical workflow and relationships supported by the current data. Dashed arrows indicate the additional experiments required before causal or stage-dependent claims can be established. Gray boxes represent findings supported by the current study, whereas red boxes represent hypotheses not established by the current data. The yellow box summarizes the key experimental approaches required for further confirmation.

Figure 14 separates what is supported from what remains untested. The supported level is an association between senescence-related transcriptomic annotations and checkpoint-related gene expression in established OA cartilage, accompanied by limited evaluation in an acute inflammatory chondrocyte model. Causal checkpoint imbalance, stage dependence, cell-cycle arrest, cellular senescence, and therapeutic efficacy remain unestablished.

### 4.6. Limitations

Several limitations materially constrain interpretation. First, the training datasets are heterogeneous: most contain bulk articular cartilage, GSE246425 contains primary cartilage-derived chondrocytes under young/replicatively aged conditions, and GSE169077 contains pooled cartilage samples. In addition, GSE98918 contains meniscal tissue and was therefore used only as a cross-tissue validation cohort. These differences limit direct tissue-specific inference. The in vitro evaluation used a human chondrocyte line, creating an additional tissue-versus-cell-model mismatch. Second, the clinical samples predominantly represent established or advanced OA, whereas 24 h IL-1β exposure is an acute inflammatory model. Direct stage comparison is therefore inappropriate.

Third, FACS-based cell-cycle analysis, p53 activation assays, CDK phosphorylation, DNA-damage markers, SA-β-gal staining, p16INK4a, SASP factors, and other senescence measurements were not performed. Thus, neither checkpoint imbalance nor cellular senescence was experimentally demonstrated. Fourth, the Western blot findings were interpreted qualitatively and were not accompanied by densitometric quantification; accordingly, protein-level statistical inference is not available. Fifth, hub selection, ceRNA/TF networks, drug enrichment, and docking are computational and may contain database and algorithm biases. Finally, there was no matched early-versus-late clinical cohort, in vivo validation, target-engagement assay, functional rescue, or prospective biomarker evaluation.

## 5. Conclusions

Senescence-associated transcriptomic changes in established OA cartilage include checkpoint-related candidates. WEE1 showed a concordant decrease at the transcript level in public cartilage datasets and IL-1β-treated C28/I2 cells, whereas CDK6 differed between clinical tissue datasets and the acute cell model. These findings are at the association-level and are hypothesis-generating. They do not prove p53-mediated CDKN1A activation, stage-dependent CDK6 regulation, G1/S-G2/M checkpoint imbalance, chondrocyte senescence, diagnostic utility, or therapeutic efficacy.

## Figures and Tables

**Table 1:** Summary of GEO datasets and their analytical roles in the present study.

Dataset	Role	Platform/Technology	Tissue/Source	Sample Composition	Relevant Characteristics
GSE57218	Training	GPL6947; Illumina HumanHT-12 v3.0	Articular cartilage	33 OA-affected/7 non-OA control used	Series also contains 33 macroscopically preserved cartilage samples matched to OA donors; these were not treated as independent non-OA controls.
GSE117999	Training	GPL20844; Agilent SurePrint G3 Human GE v3 8 × 60 K	Knee articular cartilage	12 OA/12 non-OA available	Four samples were excluded in the source study final analysis; OA samples were obtained at TKA and non-OA samples at arthroscopic partial meniscectomy.
GSE114007	Training	GPL11154/GPL18573; Illumina HiSeq 2000/NextSeq 500 RNA-seq	Human knee cartilage	20 OA/18 normal	Genome-wide RNA-seq of OA and normal knee cartilage.
GSE246425	Training	GPL24676; Illumina NovaSeq 6000 RNA-seq	Primary knee articular chondrocytes	8 OA/4 healthy profiles	Includes young and replicatively aged/passaged conditions; interpreted as cartilage-derived cellular profiles rather than bulk cartilage tissue.
GSE169077	Training	GPL96; Affymetrix Human Genome U133A	Human knee cartilage	6 OA pools/5 normal pools	Each pool contains RNA from five individual donors; pool, not individual donor, is the expression-profile unit.
GSE98918	Independent cross-tissue validation	GPL20844; Agilent SurePrint G3 Human GE v3 8 × 60 K	Meniscal tissue	12 OA/12 non-OA	Reserved exclusively for validation; not included in training, batch correction, hub selection, or model development.

**Table 2:** CB-Dock2/Vina poses for berberine with WEE1 (PDB: 8BJU).

Mode	Affinity (kcal/mol)	RMSD l.b. (Å)	RMSD u.b. (Å)
C1	−7.1	0	0
C2	−6.6	6.368	13.039
C3	−5.7	27.991	31.053
C4	−5.7	15.187	17.859
C5	−5.7	21.691	22.794

**Table 3:** CB-Dock2/Vina poses for folic acid with WEE1 (PDB: 8BJU).

Mode	Affinity (kcal/mol)	RMSD l.b. (Å)	RMSD u.b. (Å)
C1	−9.5	0	0
C2	−9.1	2.062	3.305
C3	−8.5	1.746	3.515
C4	−8.3	7.316	12.562
C5	−8.0	6.750	11.941

**Table 4:** CB-Dock2/Vina poses for berberine with CDK6 (PDB: 3NUP).

Mode	Affinity (kcal/mol)	RMSD l.b. (Å)	RMSD u.b. (Å)
C1	−8.6	0	0
C2	−8.2	1.881	7.623
C3	−7.4	1.554	8.019
C4	−6.1	22.505	23.576
C5	−6.1	24.851	26.733

**Table 5:** CB-Dock2/Vina poses for folic acid with CDK6 (PDB: 3NUP).

Mode	Affinity (kcal/mol)	RMSD l.b. (Å)	RMSD u.b. (Å)
C1	−7.1	0	0
C2	−7.0	28.489	31.662
C3	−6.9	27.716	31.193
C4	−6.7	28.002	31.169
C5	−6.6	7.952	13.070

## Data Availability

The public datasets analyzed are available in GEO under GSE57218, GSE117999, GSE114007, GSE246425, GSE169077, and GSE98918.

## Supplementary Materials

The following supporting information can be downloaded at: https://www.mdpi.com/article/10.3390/biomedicines14091914/s1, Table S1: Primer sequences used for RT-qPCR; Figure S1: Complete predicted ceRNA network for CDKN1A, CDK6, WEE1, and RBL2. Red nodes represent mRNAs, cyan nodes represent miRNAs, green nodes represent lncRNAs, and lines represent predicted interactions retained by the source-analysis criteria. Figure 8 presents a readable degree-based core subnetwork; Figure S2: Source image underlying the representative Western blot panels in Figure 13. Red rectangles in the laboratory source image identify the Control-IL-1β lane pairs selected for presentation. The source image is provided for transparency; the representative immunoblot findings are interpreted qualitatively without densitometric or protein-level inferential statistics.
