# Supplementary material for: Senescence-Associated Checkpoint Gene Dysregulation in Established Osteoarthritis: Integrated Transcriptomic Analysis and In Vitro Evaluation of CDK6 and WEE1
Source: Biomedicines. 2026 Aug 26;14(9):1914. doi: 10.3390/biomedicines14091914 (PMC13604092; doi:10.3390/biomedicines14091914)
Supplement: Supplementary file 1 [file biomedicines-14-01914-s001.zip › biomedicines-4269612-supplementary.pdf]

Supplementary Materials

Supplementary Table S1. Primer sequences used for RT-qPCR.

Gene	Forward primer (5′→3′)	Reverse primer (5′→3′)
Human WEE1	TTCTTTGTTGCTTCATTCCTC	GCAGGTCTTGTTTCATCTTCA
Human CDK6	ACGAACAGACAGAGAAACCAA	ACCTCGGAGAAGCTGAAAC
Human β-ACTIN (ACTB)	GTCATTCCAAATATGAGATGCGT	GCTATCACCTCCCCTGTGTG

S1

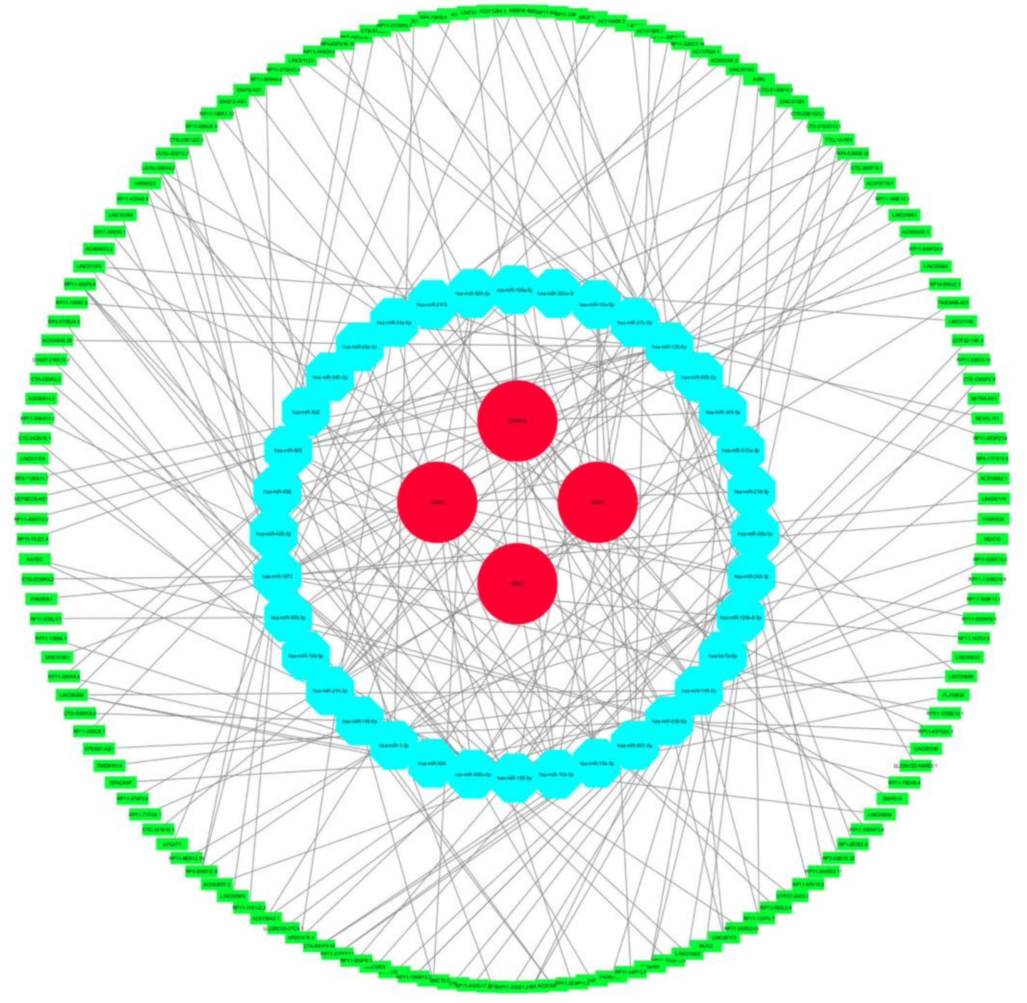

Supplementary Figure S1. Complete predicted ceRNA network for CDKN1A, CDK6, WEE1, and RBL2. Red nodes represent mRNAs, cyan nodes represent miRNAs, green nodes represent lncRNAs, and lines represent predicted interactions retained by the source-analysis criteria. Figure 8 presents a readable degree-based core subnetwork.

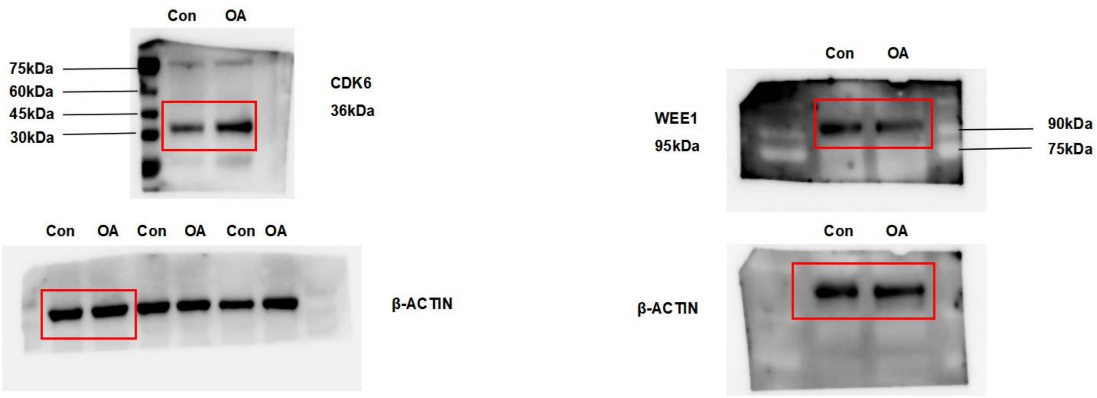

Supplementary Figure S2. Source image underlying the representative Western blot panels in Figure 13. Red rectangles in the laboratory source image identify the Control-IL-1β lane pairs selected for presentation. The source image is provided for transparency; the representative immunoblot findings are interpreted qualitatively without densitometric or protein-level inferential statistics.
